# Medical Malpractice Claims for Sports Cardiology Cases Among Young Athletes

**DOI:** 10.1016/j.jacadv.2025.101915

**Published:** 2025-06-30

**Authors:** Samantha L. Weller, Masihullah Barat, Zachary Weller, Francis Phan, Nathaniel Moulson, Timothy W. Churchill, Kimberly G. Harmon, Jonathan A. Drezner, Aaron L. Baggish, Ahmad Masri, Bradley J. Petek

**Affiliations:** a Department of Medicine, Oregon Health & Science University, Portland, Oregon, USA; b Division of Cardiology, University of California-San Diego, San Diego, California, USA; c Department of Business Administration, University of Delaware, Newark, Delaware; d Electrophysiology Service, Knight Cardiovascular Institute, Oregon Health & Science University, Portland, Oregon, USA; e Sports Cardiology Program, Knight Cardiovascular Institute, Oregon Health & Science University, Portland, Oregon, USA; f Division of Cardiology and Sports Cardiology BC, University of British Columbia, Vancouver, British Columbia, Canada; g Division of Cardiology, Massachusetts General Hospital, Boston, Massachusetts, USA; h Cardiovascular Performance Program, Massachusetts General Hospital, Boston, Massachusetts, USA; i Department of Family Medicine and Center for Sports Cardiology, University of Washington, Seattle, Washington, USA; j Swiss Olympic Medical Center, Lausanne University Hospital (CHUV), Lausanne, Switzerland; k Institute for Sport Science, University of Lausanne (ISSUL), Lausanne, Switzerland; l Hypertrophic Cardiomyopathy Center, Division of Cardiology, School of Medicine, Oregon Health & Science University, Portland, Oregon, USA

**Keywords:** athlete, exercise, lawsuit, sudden cardiac arrest, sudden cardiac death

## Abstract

**Background:**

Sudden cardiac arrest/death (SCA/D) is the leading medical cause of fatalities among young competitive athletes. Sports participation among athletes with cardiovascular disease has become more frequent, raising concerns regarding the medicolegal risk and adequacy of emergency medical response plans.

**Objectives:**

The purpose of this study was to analyze the frequency and characteristics of medical malpractice/negligence claims related to sports cardiology cases among young competitive athletes in the United States.

**Methods:**

A comprehensive retrospective review of medical malpractice/negligence lawsuits from inception to October 2024 was performed using 4 search strategies. Cases involving young competitive athletes aged 12 to 40 years competing at the middle school, high school, competitive club, collegiate, semiprofessional/professional, or national/international level who experienced SCA/D or had a diagnosis associated with SCA/D were included. Medical malpractice/negligence case frequency, location, demographics, allegations, defendant profiles, and case outcomes/awards were identified.

**Results:**

A total of 35/586 (6%) cases met inclusion criteria from 1978 to 2022. There was a favorable plaintiff outcome or settlement in 10/35 (29%) cases with known settlements or awards ranging from $600,000 to $24,000,000; a favorable defendant outcome or dismissal in 16/35 (46%) cases; and the case outcome was undisclosed/unknown in 9/35 (26%) cases. The most common primary allegation for lawsuits was a negligent emergency medical response (13/35, 37%) followed by failure to diagnose cardiovascular disease (9/35, 26%).

**Conclusions:**

Medical malpractice/negligence claims regarding cardiac cases in young competitive athletes in the United States were rare (<1 case/y), although the financial settlements were significant. This study supports ongoing efforts to improve emergency preparedness and the cardiac emergency medical response for young competitive athletes.

Sudden cardiac arrest/death (SCA/D) is the leading medical cause of death among young competitive athletes.^1^^,^^2^ Sports cardiology has emerged as a critical field for the diagnosis and management of athletes with cardiovascular (CV) disease.^3^ With the increasing prevalence of athletes with CV disease participating in competitive sports and the substantial financial resources invested in athletes within elite-level sporting leagues,^4^, ^5^, ^6^, ^7^, ^8^, ^9^, ^10^, ^11^ concerns about the medicolegal risks of providing care to this population have grown. The primary aim of this study was to analyze medical malpractice/negligence claims for sports cardiology cases among young competitive athletes in the United States.

## Methods

A comprehensive search strategy was designed to identify medical malpractice/negligence lawsuits involving young competitive athletes from inception to October 2024. Detailed search methods are presented in Supplemental Table 1. Cases were identified through 4 independent search strategies: 1) Westlaw database; 2) vLex database; 3) Westlaw/vLex/Google search for lawsuits associated with sudden cardiac death (SCD) cases from the University of Washington College Athlete Death database;^1^ and 4) cases in which authors of this manuscript served as an expert consultant. Cases were included if they involved: 1) medical malpractice/negligence lawsuits; 2) competitive athletes aged 12 to 40 years competing at the middle school, high school, competitive club, collegiate, semiprofessional/professional, or national/international level; and 3) athletes who sustained a SCA/D event or diagnosis/management of a condition associated with SCA/D per previous definitions.^4^ Cases involving exertional death related to sickle cell trait or heat stroke were excluded, as well as lawsuits filed by athletes against sporting organizations for participation restrictions due to a CV diagnosis who did not have an adverse cardiac event. Two independent reviewers (S.L.W. and M.B.) performed a full-text screen to assess for inclusion and then performed blinded data extraction. Any discrepancies during the full-text review or data extraction process were resolved by a third reviewer (B.J.P.).

Settlements and awards were adjusted for inflation using the United States Bureau of Labor Statistics Consumer Price Index Inflation Calculator (https://www.bls.gov/data/inflation_calculator.htm, date accessed May 2, 2025). The calculator converted the initial settlement/award amount to an adjusted settlement/award amount by calculating the degree of inflation from the date of the initial award/settlement to March 2025.

Data were reported using standard descriptive statistics. Categorical variables were described with frequencies (n) and percentages (%), and continuous variables were described with means and SDs. All descriptive statistics were generated using Microsoft Excel for Mac, Version 16.97 (Microsoft 365), April 2024 update. This study was deemed to be Institutional Review Board exempt.

## Results

**Figure 1:**
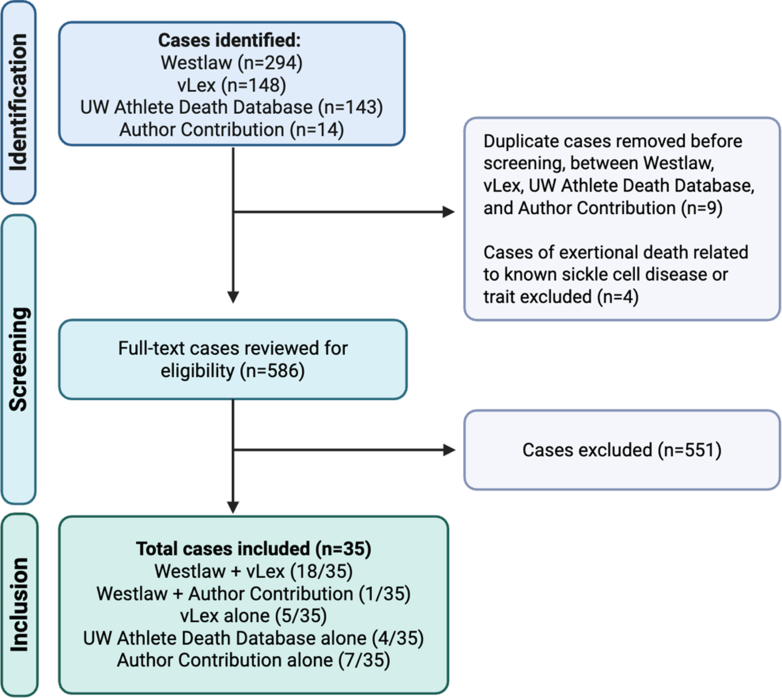
Flow Diagram for Case Identification and Inclusion UW = University of Washington.

The comprehensive search strategy yielded 586 potential cases, out of which 35 (6%) met inclusion criteria (Figure 1). The included cases ranged from 1978 to 2022 and occurred in 18 different states (Central Illustration). There was an average of 0.8 cases/y over the entire study period, and 1.8 cases/y over the last decade. The average age of athletes included was 18.9 ± 4.5 years old (range: 12-34 years old), and only 1/35 (3%) of the cases included a female athlete. Cases were most common among collegiate athletes (12/35, 34%), followed by high school (11/35, 31%), middle school (4/35, 11%), competitive club (4/35, 11%), and semiprofessional/professional athletes (4/35, 11%). The most common primary defendants were health care practitioners (Central Illustration, 15/35, 43%).

**Central Illustration:**
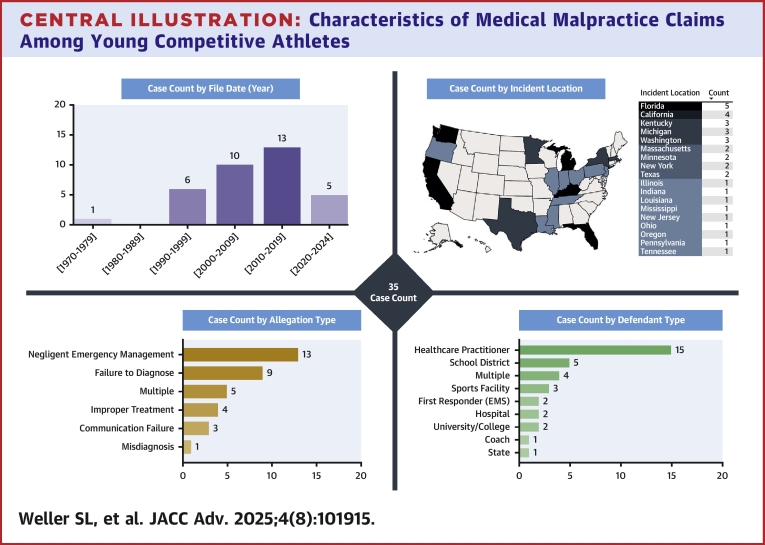
Characteristics of Medical Malpractice Claims Among Young Competitive Athletes (Top Left) Histogram of absolute case count per decade. (Top Right) Map of the United States demonstrating the number of cases per state (darker colors are associated with a higher number of cases). (Bottom Left) Bar graph of absolute case count by primary allegation type. (Bottom Right) Bar graph of absolute case count by primary defendant type.

The most common primary allegation for lawsuits was a negligent emergency medical response (13/35, 37%), followed by failure to diagnose CV disease (9/35, 26%) and multiple allegations (5/35, 14%). There was a favorable defendant outcome or case dismissal in 16/35 (46%) cases (Table 1), a favorable plaintiff outcome or settlement in 10/35 (29%) cases with settlements or awards ranging from $600,000 to $24,000,000 (Table 2) (adjusted settlements or awards ranged from $893,198 to $29,655,000), and 9/35 (26%) case outcomes were undisclosed/unknown (Table 3).

**Table 1:** Case Descriptions for Medical Malpractice and Negligence Lawsuits for Sports Cardiology Cases Involving Young Competitive Athletes With a Favorable Defendant Outcome or Dismissed Case (16/35)

Age/Sex (Year)	Allegation	Defendant	Case Description	Legal Outcome	Award Amount
12/M (1978)	Failure to diagnose	Hospital	Athlete (unspecified primary sport) suffered SCD after cleared to play by PPCS. Diagnosed with valvular heart disease as the cause of SCD on autopsy.	Favored defendant	N/A
27/M (1996)	Misdiagnosis	HCP	Basketball player with exertional syncope. Physician diagnosed neurocardiogenic syncope after tilt table test. Subsequently had SCD due to HCM.	Favored defendant	N/A
20/M (2003)	Comm failure	HCP	Failure to communicate likely HCM diagnosis by echocardiogram in basketball player who later died of SCD. PPCS was notable for heart murmur and prior syncope. Echo was ordered but athlete was cleared prior to completion.	Favored defendant	N/A
17/M (2004)	Negligent EMR	Sports facility	Failure to provide timely EMR (CPR/AED) in ice hockey player with SCA. CPR performed but AED availability was not communicated. The athlete died.	Favored defendant	N/A
21/M (2004)	Negligent EMR	Sports facility	Failure to provide timely EMR (AED) in basketball player with SCA. CPR commenced immediately by untrained bystander. No AED available. The athlete died.	Favored defendant	N/A
14/M (2006)	Negligent EMR	School district	Failure to provide timely EMR (CPR/AED) in baseball player with SCA. No CPR performed. AED available but not used. The athlete died.	Favored defendant	N/A
17/M (2008)	Failure to diagnose	HCP	Football player who suffered SCD the day after PPCS. Diagnosed with hypertensive heart disease as the cause of SCD on autopsy.	Favored defendant	N/A
34/F (2009)	Negligent EMR	EMS	Failure to provide timely EMR (CPR/AED) in softball player with SCA. EMS was called but response was delayed. CPR performed by police. The athlete died.	Dismissed	N/A
14/M (2010)	Negligent EMR	Sports facility	Failure to provide timely EMR (AED) in basketball player with SCA. CPR initiated. EMS was dispatched but unable to gain timely entry into facility. No AED available. The athlete died.	Favored defendant	N/A
17/M (2013)	Comm failure	HCP	Failure to communicate revocation of sports authorization form for athlete with SCD due to known congenital aortic valve stenosis.	Favored defendant	N/A
19/M (2013)	Negligent EMR	State	Failure to provide timely EMR (CPR/AED) in football player with SCA. CPR eventually initiated by trainer. AED available but not used until EMS arrived. The athlete died.	Favored defendant	N/A
15/M (2013)	Negligent EMR	School district	Failure to provide timely EMR (CPR/AED) in soccer player with SCA. CPR performed promptly. AED available but not used. Athlete survived with severe brain injury.	Favored defendant	N/A
16/M (2014)	Negligent EMR	School district	Failure to provide timely EMR (CPR/AED) in baseball player with SCA. No CPR performed. AED available but not used. Athlete survived with neurologic deficits.	Dismissed	N/A
15/M (2017)	Negligent EMR	Coach	Failure to provide timely EMR (CPR/AED) in basketball player with SCA. CPR was eventually commenced. AED use was significantly delayed as it was not readily available in training room.	Favored defendant	N/A
20/M (2018)	Failure to diagnose	Multiple	Failure to perform ECG during PPCS in football player who ultimately suffered SCD shortly after exercise. Diagnosed with probable cardiomyopathy as the cause of SCD on autopsy.	Dismissed	N/A
17/M (2021)	Failure to diagnose	HCP	Failure to diagnose suspected HCM in basketball player with prior exercise-related syncope. Initial echocardiogram was normal. The athlete died of SCD. Autopsy revealed septal LV hypertrophy and moderate LAD narrowing. Cause of death was highly debated by experts.	Favored defendant	N/A

AED = automated external defibrillator; Comm = communication; CPR = cardiopulmonary resuscitation; ECG = electrocardiogram; EMR = emergency medical response; EMS = emergency medical services; HCM = hypertrophic cardiomyopathy; HCP = health care practitioner; LAD = left anterior descending artery; LV = left ventricle; PPCS = preparticipation cardiovascular screening; SCA/D = sudden cardiac arrest/death.

**Table 2:** Case Descriptions for Medical Malpractice and Negligence Lawsuits for Sports Cardiology Cases Involving Young Competitive Athletes With a Favorable Plaintiff Outcome or Settlement (10/35)

Age/Sex (Year)	Allegation	Defendant	Case Description	Legal Outcome	Award Amount
23/M (1992)	Multiple	Multiple^a^	Basketball player with prior syncope, diagnosed with exercise-induced VT, suffered SCD after beta-blocker dose reduction. Autopsy revealed HCM. Allegations included misdiagnosis and negligent EMR to SCD (CPR delayed).	Settlement	$2,400,000^b^
					$5,431,830^c^
21/M (1993)	Improper treatment	HCP	Basketball player allowed to participate with ARVC and exercise-induced VT on amiodarone. Suffered SCA after dose reduction. Athlete survived with severe brain damage.	Favored plaintiff	Undisclosed
22/M (2006)	Failure to diagnose	HCP	Failure to further investigate “slight systolic murmur” detected on PPCS. Basketball player was cleared to play and later had SCD. Diagnosed with HCM on autopsy.	Favored plaintiff	$2,400,000
					$3,870,487^c^
19/M (2006)	Multiple	University	Lacrosse player with SCD. Allegations included inadequate PPCS and negligent EMR. The specifics of the allegations were not publicly disclosed. The case was privately settled.	Settlement	Undisclosed
24/M (2008)	Improper treatment	HCP	Professional baseball player had unnecessary pacemaker implantation for sinus bradycardia.	Favored plaintiff	$600,000
					$893,197^c^
15/M (2017)	Negligent EMR	School district	Failure to provide timely EMR (CPR/AED) in football player with SCA. CPR commenced immediately. No AED available. The athlete died.	Favored plaintiff	$8,000,000
					$10,453,808^c^
16/M (2019)	Negligent EMR	School district	Failure to provide timely EMR (CPR/AED) in football player with SCA. No CPR performed. AED available but not used. The athlete died.	Settlement	$5,250,000
					$6,642,025^c^
26/M (2019)	Multiple	Multiple^d^	Basketball player with “thickened heart” on echocardiogram, but no further evaluation. He suffered SCA during a game with no CPR/AED used and died in the hospital. Allegations included negligent EMR and failure to diagnose.	Settlement	Undisclosed
20/M (2020)	Multiple	HCP	Basketball player with chest pain/SOB had normal stress ECG but no further testing. He suffered SCA due to coronary artery anomaly. Delayed CPR/AED (untrained coaches) led to brain damage and permanent disability.	Favored plaintiff	$24,000,000
					$29,655,000^c^
16/M (2021)	Negligent EMR	HCP	Failure to follow EMR protocols in soccer player with SCA. CPR delayed due to lack of availability of CPR-trained staff at the time of collapse. Multiple AEDs available but not used. The athlete eventually died.	Settlement	Undisclosed

ARVC = arrhythmogenic right ventricular cardiomyopathy; SOB = shortness of breath; VT = ventricular tachycardia; other abbreviations as in Table 1.

a Defendants included university, cardiologist, and coach.

b Settlement included $1.4M from university and $1M from cardiologist.

c Settlement or award amount adjusted for inflation from year of award to March 2025.

d Defendants included sports facility, basketball company, major sport association, university board, first responder, team physician.

**Table 3:** Case Descriptions for Medical Malpractice and Negligence Lawsuits for Sports Cardiology Cases Involving Young Competitive Athletes With Undisclosed/Unknown Case Outcomes (9/35)

Age/Sex (Year)	Allegation	Defendant	Case Description	Legal Outcome	Award Amount
20/M (1992)	Negligent EMR	EMS	Failure to provide timely EMR (CPR/AED) in lacrosse player with SCA. No CPR-certified personnel present. CPR delayed until arrival of athletic trainer.	Undisclosed/Unknown	N/A
18/M (1993)	Improper treatment	Hospital	Basketball player with prior stroke of unknown cause (possible cardioembolic vs vasculitis). Cleared for sport after dose reduction of anticoagulation. Died due to additional stroke thought to be related to dose reduction.	Undisclosed/Unknown	N/A
18/M (1994)	Multiple	HCP	Failure to communicate possible diagnosis of Marfan's syndrome detected on PPCS in basketball player who later had SCD. Echo and CXR were performed but no treatment plan, referrals or risk counseling were provided.	Undisclosed/Unknown	N/A
12/M (2006)	Failure to diagnose	HCP	Failure to restrict activity and further investigate presyncope in basketball player who ultimately had SCD, later found to have anomalous coronary artery.	Undisclosed/Unknown	N/A
19/M (2010)	Comm failure	HCP	Failure to communicate likely HCM diagnosis by echo in soccer player cleared to play despite echo and detection of heart murmur on PPCS exam. The athlete later had SCD.	Undisclosed/Unknown	N/A
22/M (2018)	Failure to diagnose	Multiple	Failure to further investigate chest pain and abnormal CXR in basketball player who presented to ED for evaluation. He was discharged and later died of SCD from ruptured aortic aneurysm.	Undisclosed/Unknown	N/A
19/M (2018)	Improper treatment	University/College	Football player with multiple episodes of chest pain and dizziness during practice. Athletic trainers and coaches failed to refer for evaluation or restrict from participating in practice. The athlete suffered SCA and later died.	Undisclosed/Unknown	N/A
13/M (2020)	Failure to diagnose	HCP	Failure to properly evaluate football player who documented chest pain and exhaustion on PPCS form. Cause of death on autopsy was related to right coronary artery abnormality.	Undisclosed/Unknown	N/A
23/M (2022)	Failure to diagnose	HCP	Failure to diagnose WPW on PPCS ECG in a baseball player.	Undisclosed/Unknown	N/A

CXR = chest x-ray; ED = emergency department; WPW = Wolff-Parkinson White; other abbreviations as in Table 1.

For cases that included a negligent emergency medical response (n = 17), there were 6/17 (35%) cases with delayed cardiopulmonary resuscitation/automated external defibrillator (CPR/AED) use, 3/17 (18%) with CPR performed but no AED available, 3/17 (18%) with CPR performed and AED available but unused, 4/17 (24%) cases with no CPR/AED use despite availability, and 1/17 (6%) cases with a nonspecific allegation. There were 8/35 (23%) cases that included allegations of a failure to diagnose CV disease during or in follow-up of preparticipation CV screening. Among athletes with exertional cardiopulmonary symptoms and no prior known diagnosis, 3 cases of coronary artery anomalies leading to SCD were missed because of lack of adequate secondary testing. For athletes diagnosed with possible or definitive conditions associated with SCA/D (10/35, 29%), lawsuit allegations were related to failure to adequately communicate the diagnosis and potential risks with exercise (4/10, 40%), failure to further interrogate symptoms or abnormal testing (2/10, 20%), improper treatment (2/10, 20%), and misdiagnosis (2/10, 20%).

## Discussion

This study assessed medical malpractice/negligence claims in sports cardiology cases involving young competitive athletes in the United States, with several key findings. First, there was an average of 0.8 cases/y over the entire study period, and 1.8 cases/y over the last decade. With tens of millions of young athletes participating in competitive sports per year in the United States, the rate of sports cardiology-related medicolegal cases is exceedingly low. Second, the consequences of medicolegal claims are substantial yet highly variable, with settlements or awards ranging from $600,000 to $24,000,000 ($893,198 to $29,655,000 when adjusted for inflation). Third, young athletes presenting with exertional cardiopulmonary symptoms (especially chest discomfort) without a known diagnosis should undergo appropriate CV testing to exclude coronary artery anomalies (3 missed cases in this study). Finally, an inadequate emergency medical response was the most common cause of medical negligence/malpractice claims.

Historically, scientific statements for sports participation among young competitive athletes provided binary recommendations on whether an athlete should be “cleared” or “restricted” from competitive sports based on the underlying cardiac pathology.^12^ Multiple studies have demonstrated that vigorous exercise or competitive sports participation in certain CV conditions may carry less risk than previously expected,^5^^,^^6^^,^^8^, ^9^, ^10^ which has supported a paradigm shift in sports cardiology, advocating for a shared decision-making approach among athletes with numerous CV conditions over the recent years.^7^^,^^12^, ^13^, ^14^ In corollary, the most contemporary American College of Cardiology/American Heart Association Clinical Considerations for Competitive Sports Participation for Athletes with Cardiovascular Abnormalities, which were published in 2025,^15^ have supported a broad shared decision-making approach on return to play among athletes with CV conditions. The rise in athletes with CV conditions participating in competitive sports coupled with the financial resources invested in high-level athletics has heightened concerns about medicolegal liability for health care practitioners. While future data are needed to assess the frequency of lawsuits and types of allegations with more athletes with CV disease competing in sports, the data from the current study underscore the need to improve the emergency medical response for athletes who sustain a SCA event and the growing need for physician education in the CV screening and diagnosis of conditions at risk for SCA/D in competitive athletes.^16^^,^^17^

Multiple previous studies have demonstrated that a prompt emergency medical response can improve outcomes among individuals who sustain a SCA event.^18^^,^^19^ There have been nationwide efforts to improve the emergency medical response for athletes throughout the United States through public policy measures mandating emergency action plans, CPR training, and distribution of AEDs within close proximity to all sporting venues and practice facilities.^16^ A recent study assessing survival outcomes over 9 years among 641 young competitive athletes demonstrated that survival from SCA improved over the study period (likely from an improved emergency medical response), however, there were disparities identified with athletes of Black and other race, who were less likely to survive a SCA event when controlling for sex, level of competition, and exertional status at the time of SCA.^20^ These data are troubling and likely are due to social determinants of health and access to a prompt emergency response. Leaders in medicine need to help drive public policy on improving the emergency medical response in all sporting venues and practice facilities to improve disparities and outcomes following SCA, and this current study may further influence policymakers as there are potential legal ramifications with a negligent emergency medical response system. While the data in this manuscript and recent studies from cohorts from the United States support the urgent need to improve the emergency medical response system at the national level, ongoing efforts are needed on the international level to prevent SCD around the world.^21^

### Study Limitations

This study has limitations that warrant further discussion. First, no search strategy will universally identify all medicolegal cases (Figure 1), so the total number of cases is likely underestimated. We utilized multiple search strategies to maximize the identification of relevant cases, however, 7/35 (20%) were identified only because authors on the current study served as expert consultants, suggesting that additional similar cases may have been missed. These data highlight the importance of employing multiple strategies for case identification in future studies as a single search strategy is inadequate in capturing all cases. Second, 9/35 (26%) cases remain undisclosed/unknown. Third, the award amount was only available for 6/10 (60%) cases with a favorable plaintiff outcome or settlement. Fourth, information about medical diagnoses and treatments among athletes in cases were derived from legal documents and/or media sources rather than official medical records, introducing the potential for inaccuracies or incomplete data. Fifth, competitive athletes represent a small subset of individuals affected by sports-related SCD,^22^ therefore these data may not be universally applicable to the broader population of individuals who engage in regular exercise. Finally, this study only addressed the financial implication of medicolegal claims and not the personal and professional implications for practitioners who were sued.

## Conclusions

This study examines medical malpractice/negligence claims for sports cardiology cases among young competitive athletes in the United States. Overall, medical malpractice/negligence cases were rare, although the consequences of claims were significant. The findings in this study support ongoing efforts to improve the emergency medical response and accurate diagnosis of cardiac disease in young competitive athletes.

Perspectives **COMPETENCY IN PATIENT CARE AND SYSTEMS-BASED PRACTICE:** The current study demonstrated that the most common allegation for medical lawsuits among sports cardiology cases for young competitive athletes was a negligent emergency medical response.

**TRANSLATIONAL OUTLOOK:** These findings highlight the need to support ongoing efforts to improve the emergency medical response through enhanced education on SCA recognition, implementation of emergency action plans, CPR training, and distribution of AEDs within close proximity to all sporting venues and practice facilities.

## Funding support and author disclosures

Dr Baggish has received funding from the 10.13039/100000050National Institutes of Health/National Heart, Lung, and Blood Institute, the National Football Players Association, the 10.13039/100000968American Heart Association, and the American Medical Society for Sports Medicine to study cardiovascular outcomes among elite athletes and receives compensation for his role as team cardiologist from the U.S. Olympic Committee/U.S. Olympic Training Centers, U.S. Soccer, and U.S. Rowing. Dr Churchill has received research funding from the 10.13039/100000050National Institutes of Health/National Heart, Lung, and Blood Institute (NIH/NHLBI) and receives compensation for his role as team cardiologist for the Boston Bruins organization. Dr Drezner has received funding from the American Medical Society for Sports Medicine, the 10.13039/100000968American Heart Association, and the NCCSIR. Dr Harmon has received funding from the American Medical Society for Sports Medicine, Football Research, Inc, the Pac-12, and the 10.13039/100000968American Heart Association. Dr Masri has received research grants from Pfizer, Ionis, Attralus, and Cytokinetics, and fees from Cytokinetics, BMS, Eidos/BridgeBio, Pfizer, Ionis, Lexicon, Attralus, Alnylam, Haya, Alexion, Akros, Prothena, BioMarin, AstraZeneca, Edgewise, and Tenaya. All other authors have reported that they have no relationships relevant to the contents of this paper to disclose.
