# Supplementary material for: Medical Malpractice Claims for Sports Cardiology Cases Among Young Athletes
Source: JACC Adv. 2025 Jun 30;4(8):101915. doi: 10.1016/j.jacadv.2025.101915 (PMC12268557; doi:10.1016/j.jacadv.2025.101915)
Supplement: Supplementary data [file mmc1.docx]

**Supplemental Table 1**. Case Identification Methods and Search Strategies

| **Method of Case Identification** | **Date of Search** | **Search Strategy** |
| --- | --- | --- |
| Westlaw Database | 10/15/2024 | The following search strategy was used in the Westlaw database using the “health practitioner” filter  adv: ((Cardi! arrhythmia aort! "myocardial infarction" "heart attack" "coronary artery" "congenital heart" myocarditis commotio electrocardiogram echocardiogram "Long QT" pre-excitation Wolff-Parkinson-White WPW Brugada Kawasaki pacemaker defib! Marfan Ehlers-Danlos Loeys-Dietz "ventricular tachycardia" myopath! valv! "sudden cardiac arrest" "sudden cardiac death" "cardiac death" "cardiac arrest" "Tetralogy #of Fallot" "transposition #of the great arteries" TGA "Ebstein anomaly" pulmonic tricuspid mitral) /p (player athlet! sport! baseball basketball dance cheerleading rowing "cross country" fencing hockey football golf gymnastics lacrosse rugby soccer softball swimming skiing "track #and field" tennis volleyball "water polo" wrestling cycling marathon diving bowling) AND ("wrongful death" OR "malpractice" OR "negligence")) |
| vLex Database | 12/23/2024 | (Cardiac OR Cardiology OR Cardiologist) AND (Malpractice OR Negligent OR Wrongful) AND (athlete OR sport) |
| UW Athlete Death Database |  | A search was performed using the name of all 143 athletes with sudden cardiac death in this database in the Google search engine and Westlaw Database. The search included “athlete name” and “lawsuit” or “malpractice” to identify cases. |
| Author Expert Contribution Cases |  | All authors of the manuscript were asked if they have participated as an expert consultant in medical malpractice or medical negligence that would meet the inclusion criteria. All cases contributed by authors were identified online using the Google search engine and Westlaw Database. |
